# Black Colouration of the Knee Articular Cartilage after Spontaneously Recurrent Haemarthrosis

**DOI:** 10.1155/2016/1238392

**Published:** 2016-05-12

**Authors:** Kazu Matsumoto, Daichi Ishimaru, Hiroyasu Ogawa, Haruhiko Akiyama

**Affiliations:** Department of Orthopaedic Surgery, Gifu University School of Medicine, 1-1 Yanagido, Gifu 501-1194, Japan

## Abstract

Mild discolouration of the articular cartilage is known to gradually occur during aging. However, pathological tissue pigmentation is occasionally induced under several specific conditions. In the present case, we performed total knee replacement in a patient with recurrent haemarthrosis. However, during the operation, we observed severe black colouration of the knee articular cartilage, due to the deposition of hemosiderin and lipofuscin. To our knowledge, this is the first report of severe cartilage pigmentation, due to hemosiderin and lipofuscin deposition in articular cartilage.

## 1. Introduction

Mild discolouration of the articular cartilage is known to gradually occur during aging. However, pathological tissue pigmentation is occasionally induced under several specific conditions, including ochronotic arthritis accompanied by alkaptonuria [1–3], haemosiderosis [4], and the use of drugs for Parkinson's disease [5, 6] or antibiotics [7–9]. In the present case, we performed total knee replacement (due to osteoarthritis) in a patient with recurrent haemarthrosis. However, during the operation, we observed severe black colouration of the knee articular cartilage, due to the deposition of hemosiderin and lipofuscin after the recurrent haemarthrosis. To our knowledge, this is the first report of severe cartilage pigmentation, due to hemosiderin and lipofuscin deposition in articular cartilage, that was only caused by chronic recurrent haemarthrosis. The patient was informed that all the data from the case would be submitted for publication and gave his written consent.

## 2. Case Presentation

A 65-year-old man complained of left knee swelling and pain without any trauma episodes. His local physician performed knee aspiration, which revealed massive haemarthrosis of the knee, although the patient had no hereditary disease or drug usage that might be associated with a haemorrhagic tendency. However, despite the conservative treatment via aspiration, haemarthrosis of the knee persisted. Therefore, at 4 months after the onset, the physician performed arthroscopy, which revealed limited joint surface degeneration with no colouration, lateral meniscus degeneration, and a partial tear, which was suspected to be the source of bleeding. Thus, lateral meniscectomy was performed to address the tear.

**Figure 1:**
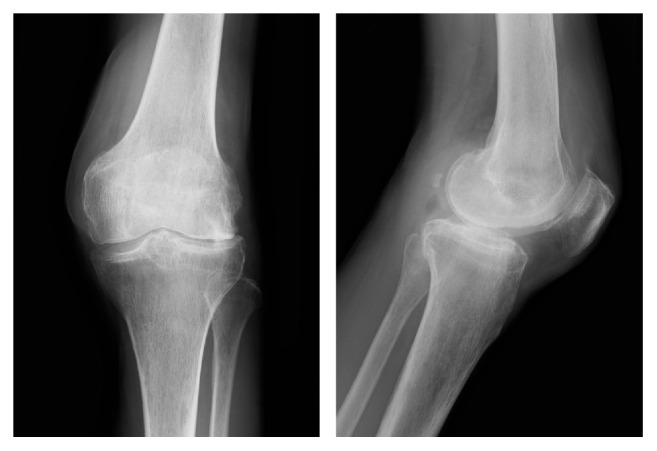
Left knee radiography at 30 months after the onset reveals progressive narrowing of the lateral joint space and osteophyte formation.

Unfortunately, the haemarthrosis of the knee persisted, and the patient was referred to our institution at 3 years after the onset, due to refractory haemarthrosis and osteoarthritis progression. At the admission, left knee radiography revealed the end stage of osteoarthritis (Figure 1), and we performed various examinations to investigate the cause of the chronic haemarthrosis. However, the cause remained unclear, as enhanced magnetic resonance imaging and thallium scintigraphy did not reveal the presence of any tumorous lesions, such as pigmented villonodular synovitis or hemangioma. Furthermore, blood analysis failed to identify the cause of bleeding tendency, as platelet counts, alanine transaminase levels, aspartate transaminase levels, activated partial thromboplastin time, and prothrombin time were all normal. As the patient did not have family history or medication that might explain the haemorrhagic tendency, we diagnosed him with spontaneous haemarthrosis and performed total knee replacement.

### 2.1. Operation

**Figure 2:**
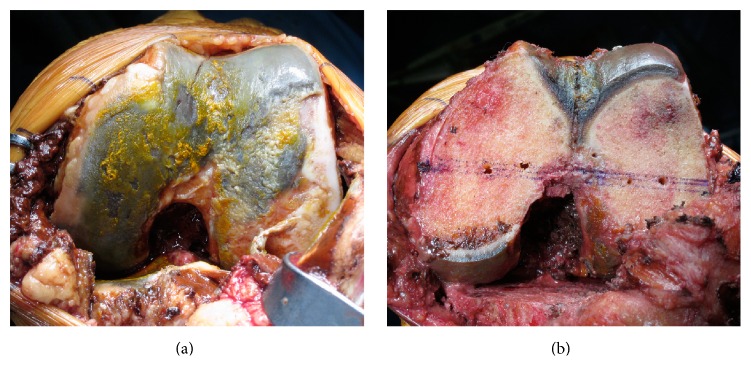
Surgical findings. (a) Macroscopic findings during total knee replacement reveal that the femoral and tibial articular cartilage exhibit brown, grey, and black pigmentation and that the synovial membranes also exhibited reddish-brown discolouration. (b) After cutting the distal femur, we observed that only the joint surface cartilage, not the subchondral bone, exhibited the black colouration.

We performed the total knee replacement under general anaesthesia using a cemented mobile-bearing prosthesis (Vanguard RP, Biomet, Japan). The articular cartilage of the left knee was exposed via the medial parapatellar approach, which revealed that the entire articular cartilage exhibited severe black pigmentation and that the synovial membrane was also pigmented (Figure 2(a)). The joint surface was eroded, and the subchondral bone was visible in part of the lateral femoral condyle. After cutting the femoral condyle to implant the prosthesis, we observed that the pigmentation was localized to the cartilage layer (Figure 2(b)). Therefore, we submitted specimens of the resected lateral femoral condyle and synovial membranes for histological examination. Despite careful evaluation, via releasing the femur tourniquet, we could not identify any obvious bleeding lesion that might have caused the chronic haemarthrosis. Nevertheless, the total knee placement was performed without complication. After the knee replacement, the patient did not experience any recurrence of the haemarthrosis over a 3-year period and reported being satisfied with the outcome, as he was able to resume playing golf.

### 2.2. Histological Examination

**Figure 3:**
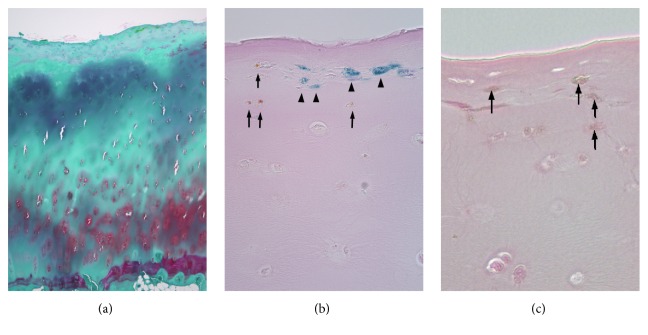
Histological findings. (a) In the lateral femoral condyle bone, safranin O staining of the cartilage lesion is reduced at the transitional and radial zones. (b) Berlin blue staining reveals hemosiderin deposition (arrowhead) in the superficial articular cartilage, and (c) lipofuscin (brown granules that are indicated by the arrows) deposition is also visible in the superficial articular cartilage via Fontana Masson staining.

Microscopic examination revealed that the synovial membranes exhibited villus structures, with partial hypertrophic change and granular pigmentation that was incorporated into the connective tissues via histiocytes. Hematoxylin and eosin staining of the distal femur cartilage revealed a noticeable decrease in the number of chondrocytes, although the normal number of chondrocytes and cartilage space were observed in the deep layer section. Double staining with safranin O and fast green revealed a remarkable reduction in the cartilage's sulphated proteoglycan content at the transitional and radial zones, which indicated articular cartilage degeneration (Figure 3(a)). Berlin blue staining confirmed iron deposition (i.e., haemosiderosis) in the joint surface cartilage (Figure 3(b), arrowheads). However, we also observed brown granulations that were negative for Berlin blue staining in the joint surface cartilage, and these granulations were stained positively using Fontana Masson stain (Figure 3(c), arrow). Therefore, based on these observations, we concluded that the cause of the severe cartilage pigmentation was the deposition of both lipofuscin and iron.

## 3. Discussion

Severe pigmentary abnormalities in the joint occasionally occur after several specific conditions, such as ochronotic arthropathy due to alkaptonuria [1–3] and the use of drugs for Parkinson's disease [5, 6] or antibiotics [7–9]. In this case, severe black colouration of the articular cartilage appears to have occurred due to haemosiderosis and lipofuscin deposition on the articular cartilage. This is the first report of specific colouration that is specific to the articular cartilage.

Ochronosis is a syndrome that is characterized by pigmentation of various connective tissues, including human cartilage, and is caused by high concentrations of homogentisic acid due to alkaptonuria (inherent homogentisic acid oxidase deficiency) [1, 2]. In addition, drug administration has been associated with cartilage pigmentation, as long-term use of levodopa and methyldopa can cause exogenous ochronosis [5, 6]. Furthermore, minocycline usage is known to cause pigmentation of the articular cartilage, although this occurs at the subchondral bone, rather than the articular cartilage [7–9]. However, our patient had no family history of alkaptonuria and was not receiving any of the drugs that are described above.

Our histological analysis revealed that iron was deposited in the extracellular matrix of the articular cartilage, which is consistent with our diagnosis of haemosiderosis. Interestingly, the hemosiderin in haemophilic arthropathy is derived from the phagocytosis of red blood cells after the formation of joint hematoma via intra-articular bleeding [10]. Unfortunately, recurrent joint bleeding (haemarthrosis) ultimately leads to a multifactorial mechanism for joint destruction. In this context, iron in the deposited blood is thought to induce the production of chemical cartilage damage mediators (e.g., neutrophil elastase, interleukin-1 alpha and beta, tumour necrosis factor alpha, calpain, and hydroxyl radicals) by inflamed synovial membrane cells, although the precise pathogenic mechanism remains unknown [11]. In our case, it appears that the recurrent haemarthrosis induced the synovitis and subsequently accelerated the joint damage. Therefore, severe cartilage pigmentation appears to indicate the end stage of joint haemosiderosis due to chronic haemarthrosis. Furthermore, haemophilic arthropathy is usually not showing the black cartilage colouration, because it causes the spontaneous haemarthrosis. Our case showed the chronic and the continuous haemarthrosis for 3 years. This specific condition, such as continuous haemarthrosis for a long time, may cause the severe black cartilage colouration.

Interestingly, severe tissue pigment abnormalities have also been caused by lipofuscin deposition. Lipofuscin is normally produced during aging, when it is formed from the oxidation of lipids and lipoproteins [12, 13], and typically presents in various tissues, including neuronal cells and the retinal pigment epithelium [14, 15]. In cartilage, lipofuscin is deposited in the costal cartilage [12] and in the nucleus pulposus of degenerated intervertebral discs [11]. However, in the present case, the recurrent haemorrhage appears to have caused knee cartilage degeneration, and lipofuscin deposition was observed in the joint's surface cartilage. Therefore, this characteristic black colouration appears to indicate both hemosiderin and lipofuscin deposition, and the combination of hemosiderin and lipofuscin might enhance the black pigmentation of joint cartilage.

In conclusion, to our best knowledge, ours is the first report of severe articular cartilage pigmentation that was caused by only chronic haemarthrosis. In this case, we conclude that the severe pigmentation and destruction of the articular cartilage (similar to that observed in ochronotic arthritis due to haemosiderosis) occurred after chronic haemarthrosis, despite the patient having no underlying disease, drug usage, or traumatic episode, which ultimately required total knee arthroplasty.
